# Biological variation and reference change value of the estimated glomerular filtration rate in humans: A systematic review and meta-analysis

**DOI:** 10.3389/fmed.2022.1009358

**Published:** 2022-10-06

**Authors:** Stefanie Thöni, Felix Keller, Sara Denicolò, Lukas Buchwinkler, Gert Mayer

**Affiliations:** Department of Internal Medicine IV (Nephrology and Hypertension), Medical University of Innsbruck, Innsbruck, Austria

**Keywords:** chronic kidney disease, CKD progression, biological variation, reference change value (RCV), estimated glomerular filtration rate

## Abstract

**Background:**

Knowledge of the biological variation of serum or plasma creatinine (Cr) and the estimated glomerular filtration rate (eGFR) is important for understanding disease dynamics in Chronic Kidney Disease (CKD). The aim of our study was to determine the magnitude of random fluctuation of eGFR by determining its reference change value (RCV).

**Methods:**

We performed a systematic review and meta-analysis of studies on biological variation of Cr. Relevant studies were identified by systematic literature search on PubMed. Additional studies were retrieved from the European Federation of Clinical Chemistry and Laboratory Medicine (EFLM) Biological Variation Database. Random-effects meta-analysis was conducted to derive an overall estimate of intra-individual variation of creatinine (CV_ICr_). Based on our estimate of CV_ICr_ and RCV for Cr, the RCV for the eGFR was determined.

**Results:**

Among identified studies, 37 met our inclusion criteria. Meta-analysis of all studies yielded a CV_ICr_ of 5.2% (95% confidence interval [CI] 4.6–5.8%), however high between-study heterogeneity (*I*^2^ = 82.3%) was found. Exclusion of outliers led to a significant reduction of heterogeneity while still including 85% of all studies and resulted in a slightly lower CV_ICr_ of 5.0% (95% CI 4.7–5.4%). Assuming an analytical variation of CV_A_ 1.1%, we found an overall RCV for eGFR of ±16.5%. After exclusion of outlier studies, we found a minimum conservative RCV for eGFR of ±12.5%.

**Conclusion:**

The RCV of the eGFR represents a valuable tool for clinicians to discern true changes in kidney function from random fluctuation.

## Introduction

Serum or plasma creatinine (Cr), along with the estimated glomerular filtration rate (eGFR), is the most commonly used marker of kidney function. In everyday clinical practice, diagnostic and therapeutic decisions are based on changes in serially determined eGFR (1). According to the KDIGO Clinical Practice Guidelines for the Evaluation and Management of Chronic Kidney Disease (CKD), eGFR and albuminuria should be assessed at least annually in patients with CKD and even more frequently in individuals with a high risk of progression (2). Based on these serial measurements of eGFR, clinicians should draw conclusions on initiation or change of treatment, yet, there is considerable controversy as to what constitutes a significant change in eGFR.

Due to biological variation, serially determined eGFR may vary without necessarily corresponding to an actual change in kidney function. Therefore, an understanding of the magnitude of the biological variation of eGFR is essential for a correct interpretation of serial test results in order to discern true changes in kidney function from reversible fluctuation (3). Since eGFR is estimated based on Cr, biological variation of Cr is directly reflected in the biological variation of eGFR.

There are many sources of variation in the process of generating laboratory test results. The main sources of variation can be attributed to preanalytical, analytical (CV_A_) and intra-individual variation (CV_I_). In real-life setting, pre-analytical variation accounts for the largest part of biological variation, however, its contribution is considered negligible in the context of clinical studies where samples are collected and handled under standardized conditions. CV_A_ occurs during the analysis of the sample and is associated with the analytical accuracy and precision. To meet analytical quality specifications CV_A_ must be strictly controlled by clinical laboratories, but unlike preanalytical variation, it cannot be completely avoided. CV_I_ represents physiological fluctuations of the analytes concentration around the individual’s homeostatic set point in a steady state condition. CV_I_ is considered to be random and the homeostatic set point varies between individuals (4).

Knowledge about the magnitude of biological variation is important, as only changes exceeding this variation can be considered a true change in kidney function. This concept is formally expressed in the notion of the reference change value (RCV), a statistic originally derived by Harris et al. (5) and further developed by Fraser et al. (6). A 95% RCV represents the smallest difference of two serial results from the same individual that cannot be explained by the underlying CV_I_ and CV_A_ of the analyte of interest. Thus, the 95% RCV marks the limit of change needed to exceed inherent biological variation, allowing a type I error of 5% (7).

The aim of this study was to determine the magnitude of biological variation of eGFR in humans to discern reversible physiological fluctuation from true deterioration or improvement of kidney function. In addition, we aimed to investigate the impact of CKD on biological variation. For this purpose, we systematically reviewed literature on biological variation of Cr and calculated the RCV of eGFR based on reported estimates of CV_I_.

## Materials and methods

This systematic review was conducted in accordance with the Preferred Reporting Items for Systematic Review and Meta-Analyses (PRISMA).

### Data sources and search strategy

PubMed was searched for literature on biological variation of Cr up to November 18th, 2021.

For the electronic literature search a combination of keywords including (“creatinine” OR “estimated glomerular filtration rate”) AND (“biological variability”) was employed. The complete search strategy is provided in the Supplementary materials. No restrictions on language, publication year or article type was made. In addition, publications on biological variation were retrieved from the European Federation of Clinical Chemistry and Laboratory Medicine (EFLM) Biological Variation Database (8) using the search term “creatinine.” Finally, reference lists of relevant articles were manually reviewed for any additional information.

### Study selection and eligibility criteria

The complete search results were merged and duplicates were removed using Endnote. Next, titles and abstracts were scanned by one reviewer. If the abstract was not informative, we retrieved the full text to check eligibility. During initial screening animal studies, studies in pediatric patients and studies not related to the aim of this review were excluded.

To assess methodological quality of the identified publications, they were then appraised and graded by the biological variation data critical appraisal checklist (BIVAC) (9). The checklist was published by the EFLM in 2018 and was designed to enable critical assessment of existing literature on biological variation data. The BIVAC is based on 14 quality items, each of which can be rated A, B, C, or D, indicating decreasing compliance. An overall BIVAC score is set based on the lowest grade achieved in any of the 14 items. For instance, if the lowest grade in any of the 14 quality items is C, the overall BIVAC score is C. For publications already appraised by EFLM Biological Variation Database (8), the provided score was adopted; publications not listed by the EFLM were assessed by two independent reviewers using the BIVAC. Any disagreement regarding the BIVAC score was resolved by reaching consensus between the two reviewers and referral to a third author for adjudication. Studies were excluded if they met any of the following exclusion criteria: (i) retrospective study design, (ii) biological variation data is estimated based on less than three samples per study participant, (iii) studies with an overall BIVAC grade D.

### Data extraction

Data was extracted by one reviewer using a standardized data collection form. Among other variables, data on publication year, study design, number of subjects, age, sex, health status, study duration, sampling intervals, samples per patient, CV_I_, and CV_A_ was extracted.

### Statistical analysis

#### Derivation of the reference change value of estimated glomerular filtration rate

To derive the RCV of eGFR, we first performed a meta-analysis of CV_*ICr*_. This overall estimate of intra-individual variation was then used to calculate the RCV of Cr, which we then used to derive the RCV of eGFR. A similar approach was shown by Badrick et al. (10), who transform the CV_*ICr*_ to the CV_I_ of the eGFR before calculating the RCV of eGFR. As eGFR is estimated and not measured, no CV_A_ for eGFR is available. In contrast to Badrick et al. (10) our approach does not assume a value of CV_A_, but rather transforms the RCV of Cr, for which a measure of CV_A_ is available. For both equations, MDRD and CKD-EPI, the only parameter subject to physiological fluctuation is Cr. The relationship between eGFR and Cr is described by a power function. Therefore, the magnitude of the exponent of Cr directly represents the magnitude of a relative change in eGFR as response to a relative change in Cr (11). Due to this relation between eGFR and Cr, the RCV of eGFR corresponds to the RCV of Cr in the same fashion. Following the equation, a relative change of Cr beyond physiological limits proxies a relative change of eGFR beyond physiological limits.

Based on the MDRD equation, a 1% change in Cr ceteris paribus corresponds to a 1.154% change of the eGFR. In contrast to the MDRD equation, the CKD-EPI equation has different exponents for a given value of Cr, depending on whether it is greater or lower than 0.7 mg/dl in women and 0.9 mg/dl in men. Therefore, assessment of the RCV based on the CKD-EPI equation leads to four different RCVs depending on sex and Cr value. Compared to measured GFR, the CKD-EPI equation performs better than the MDRD equation, especially at higher eGFR. However, as the focus of this analysis is on assessment of progression, i.e., a change in eGFR, rather than a static measurement, our analyses are performed based on the MDRD equation delivering one RCV independent of sex and Cr.

For calculation of the RCV, a CV_A_ of 1.1% was assumed. This value for CV_A_ was derived from the routine laboratory of the Medical University in Innsbruck for the enzymatic method. The value is in line with CV_A_ values reported by recent literature and corresponds to today’s analytical performance (12–14).

If the observed measurand follows a normal distribution, a symmetric, bidirectional RCV can be calculated. If the observed measurand follows a right-skewed distribution, separate limits for positive and negative changes might be more appropriate (15).

The MDRD and CKD-EPI equations for estimating eGFR, as well as the RCV formula are shown in Table 1.

**TABLE 1:** Different equations for estimated glomerular filtration rate (eGFR) and RCV equation.

*MDRD* (37)	*GFR* = 175*x*(*Cr*)^−1.154^*x*(*Age*)^−0.203^*x*0.742 (*if female*)*x*1.212(*if African American*)		

		*Serum creatinine mg/dl*	
	*female*	*≤0.7*	*GFR* = 144 × (*Cr*/0.7)^−0.329^ × (0.993)^*Age*^
*CKD-EPI* (38)		*>0.7*	*GFR* = 144 × (*Cr*/0.7)^−1.209^ × (0.993)^*Age*^
	*male*	*≤0.9*	*GFR* = 144 × (*Cr*/0.9)^−0.411^ × (0.993)^Age^
		*>0.9*	*GFR* = 144 × (*Cr*/0.9)^−1.209^ × (0.993)^*Age*^
	$\sigma =\sqrt{l\,n\,\left({\left(C\,V_{I}+C\,V_{A}\right)}^{2}+1\right)}$		

*RCV* (15)	${\text{RCV}}_{p\,o\,s}\left(\%\right)=100\%\times \left[exp\left(z\times \sqrt{2}\times \sigma \right)-1\right]$		
	$RCV_{\text{neg}}\left(\%\right)=100\%\times \left[exp\left(z\times \sqrt{2}\times \sigma \right)-1\right]$		

Z is the number of standard deviations corresponding to the desired significance level for detecting differences. For a 5% significance level, we used 1.96 as the appropriate Z-score for bidirectional change.

#### Meta-analysis and meta-regression

Due to differences between studies, a random effects meta-analysis with inverse variance weighting was performed. As inference about coefficients of variation is not common, standard errors for this statistics were often not reported. Thus, to gain equally comparable weights for each study, we used approximations of the standard errors of all CV_I_s, i.e., the CV_I*Cr*_ divided by the square root of two times the number of samples employed in estimation (16).

Studies that analyzed and compared different subgroups (i.e., different age groups or different health status) and thus reported biological variation data for each subgroup, were treated as separate studies in our meta-analysis. Different subgroups of the same study are marked with a number in square brackets after the author’s name.

We expected heterogeneity induced by age, sex, study duration, sampling interval, and health status. Though sample size is fairly small, we ran respective univariate and multivariate random effects meta-regressions.

In addition, we performed a subgroup analysis for CKD status.

#### Outlier analysis

Outliers analysis was performed following Viechtbauer and Cheung (17). Studies that showed significantly high studentized residuals, i.e., a significant deviation from the pooled overall estimate, were removed.

Statistical analysis was conducted using R, version 4.2.1 (18). For meta-analysis the package meta in version 5.2-0 was used.

## Results

**FIGURE 1:**
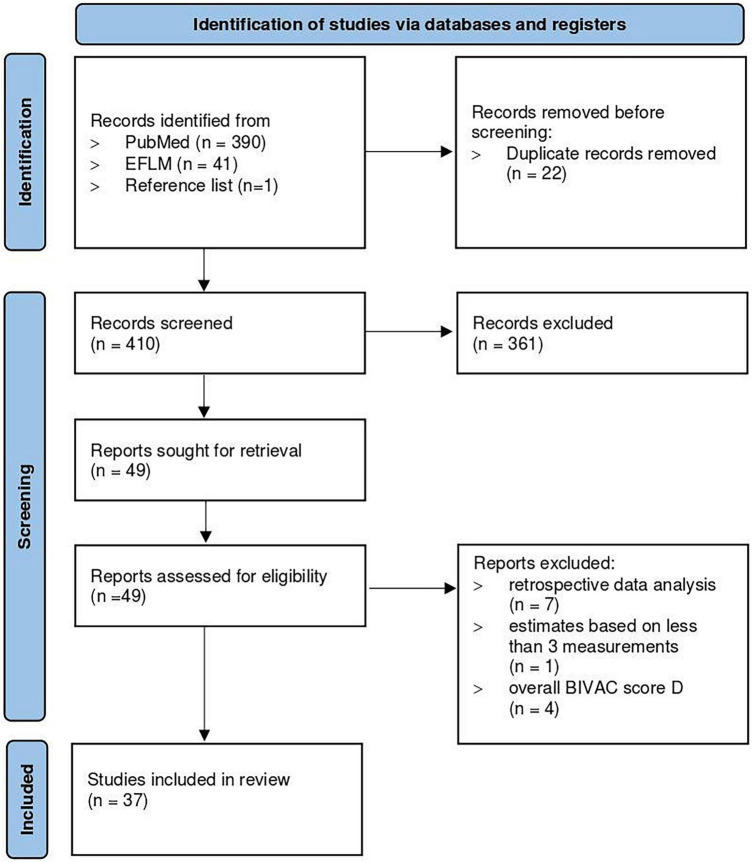
Flowchart of the study selection process. The image is adapted from Page et al. (30).

Our systematic review on biological variation of Cr yielded 390 publications on PubMed and 41 publications on the EFLM Biological Variation Database. One additional study was retrieved via screening of the reference lists. After removal of duplicates and exclusion of studies not related to the aim of our review, 49 publications were left for quality assessment by the BIVAC. Of these, 12 additional studies met the exclusion criteria leaving 37 studies for analysis. Hilderink et al. (14), Meijers et al. (19) and Reinhard et al. (20) reported biological variation data separately for healthy vs. non-healthy study groups and are therefore each analyzed separately. Similarly, Carobene et al. (21), Larsson et al. (22), Hölzel et al. (23) and Pineda-Tenor et al. (24) compared different groups and were therefore each analyzed as two, three, or four separate subgroups. In total, 37 studies and 48 different subgroups with 2,770 participants were analyzed. A flowchart of the study selection process is presented in Figure 1.

Studies included in the present review were published between 1971 and 2021, the sample sizes ranged from 2 to 1,105 participants. A total of 19 studies (51%) measured creatinine by Jaffe method, 8 studies (22%) by the enzymatic method. Sampling intervals differed among the studies, ranging from hourly to daily, weekly, and monthly sampling. On average, studies achieved an overall BIVAC score C, only 4 (11%) and 3 (8%) studies were rated with an overall BIVAC score A or B, respectively. A total of 25 (68%) studies were conducted in healthy study participants, 9 (24%) in non-healthy study participants, and 3 (8%) studies compared healthy vs. non-healthy study participants. For 6 (12%) studies the presence of CKD was specified. CV_I_ ranged from 1.2 to 13.4%, CV_A_ ranged from 0.6 to 6.7%.

The main study characteristics are shown in Table 2. Additional information on study characteristics are provided in the Supplementary material.

**TABLE 2:** Main characteristics of included studies.

Study (author and year of publication)	Number of subjects	Average Age	Number of samples per study participant	Health status	Analytical method	Overall BIVAC score	CV_I_ (%)	CV_A_ (95%-CI)
Bandaranayake et al. 2007 (39)	10	21	6	healthy	Jaffe	C	6.1	2.3 (2.0, 2.8)
Baysoy et al. 2021 (34)	22	44	10	healthy	Jaffe	A	3.3	5.56 (5.1, 6.1)
Biosca et al. 2006 (26)	19	50	33	non healthy	Unknown	C	11.8	1
Biosca et al. 1997 (40)	40	42	8	non healthy	Jaffe	C	8.5	5.4 (4.6, 6.6)
Carobene et al. 2017 (13)	8	45	4	healthy	Jaffe	C	7.8	1.1 (1.1, 1.2)
Carobene et al. 2012 [1] (21)	9	84	4	healthy	Jaffe	C	8.0	n.a.
Carobene et al. 2012 [2] (21)	13	28	4	healthy	Jaffe	C	3.8	n.a.
Carobene et al. 2012 [3] (21)	91	52	10	healthy	Enzymatic	A	4.4	n.a.
Carter et al. 2016 (41)	80	68	6	non healthy	Enzymatic	B	5.7	0.6 (0.6, 0.6)
Costongs et al. 1985 (42)	274	41	6	healthy	Unknown	C	5.7	1.6 (1.4, 1.8)
Dimitri et al. 1992 (43)	5	28	10	healthy	Jaffe	C	4.3	2.9 (2.4, 3.6)
Fraser et al. 1983 (44)	9	n.a.	14	non healthy	Jaffe	C	6.4	2 (1.8, 2.3.)
Fraser et al. 1989 (45)	27	77	10	healthy	Unknown	C	4.3	2.8 (2.6, 3.1)
Fraser et al. 1982 (27)	20	43	31	healthy	Jaffe	C	13.4	4.8 (4.6, 5.1)
Gallagher et al. 1992 (46)	5	31	5	healthy	Jaffe	C	8.4	3.2 (2.5, 4.4)
González-Revaldería et al. 1991 (47)	15	n.a.	4	Healthy	Jaffe	C	6.0	2.25 (1.9, 2.7)
Gowans et al. 1988 (48)	15	37	10	healthy	Jaffe	C	4.1	3.4 (3.1, 3.8)
Hilderink et al. 2018 [1] (14)	17	72	24	healthy	Enzymatic	A	6.4	1.1 (1.0, 1.3)
Hilderink et al. 2018 [2] (14)	19	66	24	non healthy	Enzymatic	A	2.5	1.3 (1.1, 1.5)
Hölzel et al. 1987 [1] (23)	10	41	8	healthy	Jaffe	C	2.6	3.3 (2.9, 3.9)
Hölzel et al. 1987 [2] (23)	14	27	8	healthy	Jaffe	C	2.8	3.3 (2.9, 3.8)
Hölzel et al. 1987 [3] (23)	17	n.a.	8	non healthy	Jaffe	C	5.3	3.3 (2.4, 5.3)
Keevil et al. 1998 (49)	12	40	10	healthy	Jaffe	C	4.9	3.1 (2.8, 3.6)
Larsson et al. 2009 [1] (22)	7	25	48	healthy	Jaffe	C	4.2	3.2
Larsson et al. 2009 [2] (22)	7	25	48	healthy	Jaffe	C	4.3	3.2
Matsubara et al. 2008 (50)	135	41	11	healthy	Unknown	C	6.2	2.6
Meijers et al. 2017 [1] (19)	28	43	5	healthy	Jaffe	C	4.1	1.6 (1.2, 2.5)
Meijers et al. 2017 [2] (19)	83	64	3	non healthy	Jaffe	C	5.0	1.6 (1.3, 2.2)
Nunes et al. 2010 (51)	56	18	4	healthy	Unknown	B	8.5	4.6 (3.6, 6.5)
Ozturk et al. 2013 (28)	70	46	6	non healthy	Jaffe	B	9.2	3.5
Pineda-Tenor et al. 2013 [1] (24)	56	34	4	healthy	Jaffe	C	4.9	2.6 (2.1, 3.5)
Pineda-Tenor et al. 2013 [2] (24)	62	34	4	healthy	Jaffe	C	5.0	2.6 (2.0, 3.6)
Pineda-Tenor et al. 2013 [3] (24)	64	86	4	healthy	Jaffe	C	7.0	2.6 (2.0, 3.6)
Pineda-Tenor et al. 2013 [4] (24)	71	85	4	healthy	Jaffe	C	7.1	2.6 (2.1, 3.5)
Qi et al. 2015 (52)	40	45	5	healthy	Unknown	C	4.3	1.6 (1.5, 1.8)
Ravn et al. 2016 (53)	28	62	14	non healthy	Unknown	C	3.7	n.a.
Reinhard et al. 2009 [1] (20)	19	61	8	non healthy	Enzymatic	C	8.9	1.4 (1.3, 1.6)
Reinhard et al. 2009 [2] (20)	20	44	8	healthy	Enzymatic	C	4.7	1.6 (1.4, 1.8)
Rosano et al. 1982 (54)	2	44	24	healthy	Jaffe	C	7.9	6.7 (5.6, 8.4)
Rowe et al. 2019 (12)	20	71	4	non healthy	Enzymatic	A	4.4	0.7 (0.6, 0.8)
Statland et al. 1973 (55)	11	24	3	healthy	Jaffe	C	5.4	2.9 (2.3, 3.8)
Toffaletti et al. 2008 (56)	30	44	6	healthy	Enzymatic	C	5.8	n.a.
Waikar et al. 2018 (57)	50	57	3	non healthy	Enzymatic	C	5.4	n.a.
Wang et al. 2021 (58)	25	36	6	healthy	Enzymatic	C	4.3	1.4
Williams et al. 1978 (59)	1,105	n.a.	5	healthy	Unknown	C	5.4	4.9
Winkel et al. 1974 (29)	11	24	5	healthy	Unknown	C	3.7	3.4
Winkel et al. 1976 (29)	10	26	6	healthy	Unknown	C	1.2	n.a.
Young et al. 1971 (60)	9	31	10	healthy	Jaffe	C	4.4	3.2 (2.8, 3.8)

n.a. not available.

**FIGURE 2:**
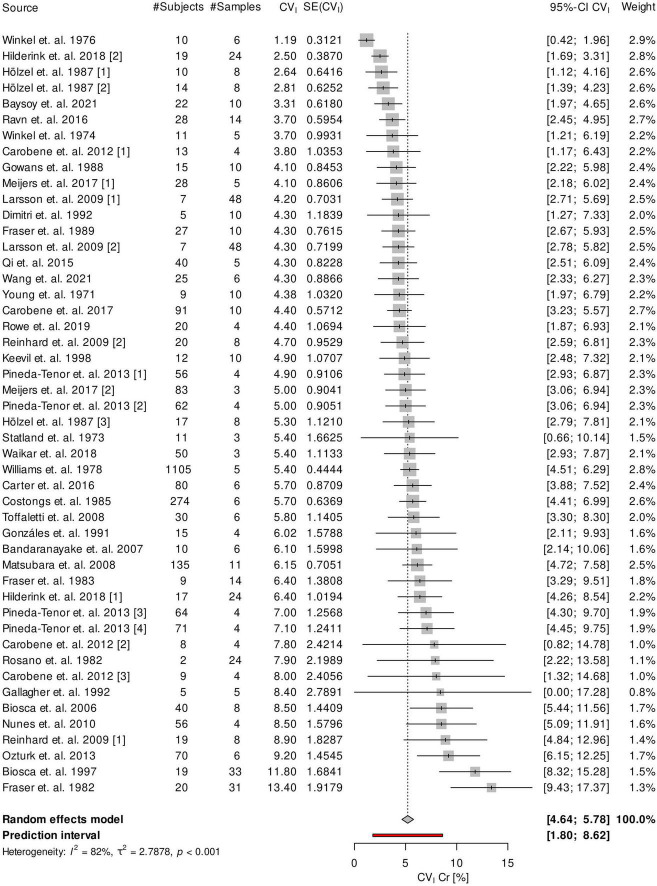
Forest plot of the meta-analysis of CV_I_ of creatinine. SE, standard error.

Analysis of the 37 included studies showed a high between-study heterogeneity (*I*^2^ = 82.3%). Meta- analysis of all studies yielded an overall CV_I*Cr*_ of 5.2% (95% confidence Interval [CI], 4.6–5.8%). Heterogeneity is also reflected in a rather large 95% prediction interval ranging from 1.8 to 8.6% (25). A Forest-Plot of the meta-analysis is shown in Figure 2.

By analysis of outliers we found the studies of Biosca et al. (26), Fraser et al. (27), Hilderink et al. (14), Hölzel et al. (23), Ozturk et al. (28), and Winkel et al. (29) to significantly increase heterogeneity. Exclusion of these studies lead to a considerable decrease of heterogeneity (*I*^2^ = 34.2%) while only slightly decreasing the overall estimate of CV_I*Cr*_ to 5.0% (95% CI, 4.7–5.4%). Exclusion of outliers led to a narrower 95% prediction interval ranging from 3.8 to 6.3%.

CV_I*Cr*_ tended to be higher in subjects affected by CKD but no significant difference between the two groups was found. Subgroup analysis for the presence of CKD is shown in the Supplementary material.

No significant impact of age, sex, CKD status, analytical method, study duration, and sampling interval on the CV_I*Cr*_ was found, hinting toward heterogeneous groups. CV_A_ tended to decrease over time showing lower CV_A_ values in more recent publications. On average, CV_A_ was lower for the enzymatic method as compared to the Jaffe method. Since CV_I_ is derived using ANOVA methods, it is by design separated and unaffected by CV_A_.

Based on the CV_I*Cr*_ at the lower end of our prediction interval after exclusion of outliers, we found a minimum conservative RCV for eGFR of ±12.5%.

Based on all included studies before exclusion of outliers, we found an overall RCV for eGFR of ±16.5%.

**FIGURE 3:**
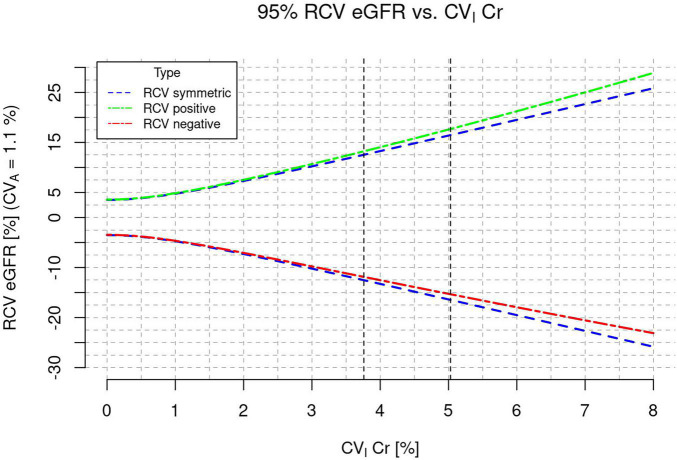
Reference change value (RCV) of estimated glomerular filtration rate (eGFR) as a function of CV_I_ of creatinine.

As seen in Figure 3, the overall symmetric RCV and the unidirectional, asymmetric RCV only differed slightly.

## Discussion

Estimated glomerular filtration rate is used in everyday clinical practice as a marker of excretory kidney function. Especially in patients with CKD, where regular assessment of the eGFR is required, a correct interpretation of serial changes in eGFR is crucial for understanding disease dynamics and for clinical decision making.

Based on the present systematic review, we found a minimum conservative RCV of 12.5%. This means, that for a patient with a baseline eGFR of 60 ml/min/1.73 m^2^ a change to an eGFR >68 ml/min/1.73 m^2^ or <53 ml/min/1.73 m^2^ represents a true improvement or deterioration of kidney function. By definition, changes within this range would have a 95% probability of being due to biological variation. For the overall RCV estimate of 16.5% the same applies to an eGFR range of 50–70 ml/min/1.73 m^2^.

Assuming the KDIGO definition of CKD progression with a 25% drop in eGFR (31), only a change to an eGFR <45 ml/min/1.73 m^2^ would imply disease progression.

This shows how true deterioration of kidney function may occur at smaller changes than currently defined.

To date, there is considerable controversy as to what constitutes a progression of CKD and different definitions can be found in literature (31, 32). At the same time, the availability of evidence-based interventions to slow CKD progression highlights the importance of an early recognition of kidney function decline (33).

The implementation of the RCV as an additional decision making tool for clinicians has been suggested before (34). Especially in the context of monitoring disease dynamics, the use of the RCV rather than a population-based reference interval seems reasonable. While population-based reference intervals (RI) reflect the variation around the homeostatic set point of different individuals, also known as the between-subject biological variation (CV_*G*_), the RCV reflects the variation around one individual’s homeostatic set point (CV_I_), i.e., the within-subject biological variation. This explains why the use of the RCV would represent a step toward personalized medicine.

For laboratory parameters with a high index of individuality (II), defined as II = CV_I_/CV_*G*_, and especially for an II >1.4, the use of RIs is considered suitable. For laboratory parameters with a low II, i.e., when CV_I_ is lower compared to CV_*G*_, the individual itself, rather than the reference population, is considered to be the best point of reference for the assessment of serial change (35). With an II of 0.3 (12), the latter also applies to the eGFR.

The high CV_*G*_ (12, 14) of eGFR is also reflected in highly heterogeneous patterns of disease progression in patients with CKD, and explains why finding a uniform definition of CKD progression is challenging and may not be suitable for everyone.

This was also shown by Kerschbaum et al. (36) who analyzed the longitudinal eGFR trajectory of patients with diabetic kidney disease by applying various definitions of CKD progression. The allocation of patients to groups with a certain confirmed drop of eGFR (e.g., ≥25, ≥30, ≥35 or ≥40%) did not result in similar patterns of disease progression over time. Next to the high variation of disease progression between subjects, also non-linearity of eGFR trajectories was frequently observed. For example, in patients with a confirmed eGFR drop of >30% only 60.3% and 45.2% lost at least the same amount between baseline and year 4 or 5. The remainder did not show a disease progression but rather a recovery of kidney function. This behavior was also shown for patients on stable medication (36).

The overall CV_ICr_ reported by our study is in broad agreement with the overall estimate of the EFLM Biological Variation Database (CV_I_ 4.4% [95% CI 4.2–5.7%]) (8). Similarly, our overall RCV is in line with previously published RCVs (12, 14).

Subgroup analysis by the presence of CKD tended to show higher CV_I_ values in subjects affected by CKD, however, literature on this behalf is discrepant: In 2018, Hilderink et al. (14) found a significantly higher CV_ICr_ in subjects without CKD (CV_ICr_ 6.4%, mean eGFR 73.4 ± 18.5 ml/min/1.73 m^2^) compared to subjects with CKD (CV_ICr_ 2.5%, mean eGFR 19.2 ± 6.4 ml/min/1.73 m^2^) (14). Reinhard et al. (20) had previously compared healthy subjects to subjects with mild to moderate CKD. In this case, although not significantly, CV_ICr_ was almost twice as high in the CKD group compared to the healthy group (8.9 vs. 4.7%) (20).

The main limitation of our meta-analysis is the high between-study heterogeneity. Nevertheless, after exclusion of seven studies, we see a marked reduction of heterogeneity while still including 85% of all studies. An important driver of heterogeneity may be the health status of the study populations. While subjects classified as healthy form a more homogeneous group, non-healthy study populations included patients affected by a variety of different conditions.

Although choosing more stringent eligibility criteria would have had advantages in terms of heterogeneity, our approach aimed at investigating the impact of different variables on biological variation. Knowledge on how biological variation changes in relation to kidney function is of great importance from a clinical perspective. Analysis by health status as well as by presence of CKD showed no significant impact on biological variation in our analysis, however, our analysis was limited by a small sample size. Altogether, literature on this behalf remains inconclusive. We do believe that an influence of health and disease on biological variation is likely–especially for CKD–and should therefore be subject of further research. Despite the between-study heterogeneity, our estimate of CV_*ICr*_ is comparable to the one reported by the EFLM (8).

Our analysis is based on the MDRD equation. This seemed the more feasible approach, as the exponent of Cr is independent of sex and level of Cr. Besides, the aim of our work is to determine the significance of change of the eGFR, rather than to determine the eGFR, for which the formula by CKD-EPI has shown to be more accurate. Technically, the RCV can also be derived based on the CKD-EPI formula. However, given the different exponents of Cr, the use of the CKD-EPI equation implies that RCV is lower in subjects with higher eGFR and vice versa. As discussed above, there is limited knowledge on how biological variation changes according to GFR, thus, the use of the MDRD seemed more appropriate.

Our analysis included studies with different analytical methods for the measurement of Cr. Since CV_I_ is derived using ANOVA methods, it is by design separated and unaffected by CV_A_. However, the greater imprecision of the Jaffe method delivers higher values of CV_A_ and thus higher RCVs. If RCVs are applied to monitor kidney function in a clinical context, knowledge on the analytical method by which creatinine is measured is required.

Data on biological variation reported here were collected under idealized and standardized conditions.

This is also required by Quality Item 5 of the BIVAC to ensure that estimates of CV_I_ are not affected by preanalytical variation. According to the BIVAC, authors are encouraged to provide information on preanalytical procedures and to follow a standardized protocol (9). For instance, in many of our studies, blood samples were drawn by the same investigator from fasting patients at the same time of the day. The samples were then further processed and analyzed in the same, standardized manner. If preanalytical procedures are not standardized, increased preanalytical variation could lead to an overestimation of CV_I_. Even though many procedures (i.e., test ordering, patient preparation, specimen collection and specimen processing, transportation, and storage) may also follow a standardized protocol in clinical routine, they are more difficult to monitor outside of clinical studies. Therefore, it is likely that the CV_ICr_, and therefore the RCV, is higher in real-life settings.

## Conclusion

Knowledge about the biological variation of the eGFR is essential for understanding disease dynamics and monitoring kidney function. The RCV provides a valuable tool for clinicians to interpret changes in serial eGFR, however, more studies on biological variation in CKD need to be performed to understand how impairment of kidney function affects biological variation and if higher biological variation is associated with disease progression.

## Data availability statement

The raw data supporting the conclusions of this article will be made available by the authors, without undue reservation.

## Author contributions

ST did the systematic literature research and wrote the first draft of the manuscript. FK conceived and conducted the statistical analyses. ST, LB, and FK scored and assessed included studies. ST, FK, LB, SD, and GM reviewed and approved the final version of the manuscript.

## Supplementary material

Supplementary Image 1 Subgroup analysis for presence of CKD.

Supplementary Table 2 Main study characteristics.

## References

[1] Kashani K Rosner MH Ostermann M. Creatinine: from physiology to clinical application. *Eur J Intern Med.* (2020) 72:9–14. 10.1016/j.ejim.2019.10.025 31708357

[2] Inker LA Astor BC Fox CH Isakova T Lash JP Peralta CA KDOQI US commentary on the 2012 KDIGO clinical practice guideline for the evaluation and management of CKD. *Am J Kidney Dis.* (2014) 63:713–35. 10.1053/j.ajkd.2014.01.416 24647050

[3] Fraser CG. Biological variation: a rapidly evolving aspect of laboratory medicine. *J Lab Precis Med.* (2017) 2:35. 10.21037/jlpm.2017.06.09

[4] Braga F Panteghini M. Generation of data on within-subject biological variation in laboratory medicine: an update. *Crit Rev Clin Lab Sci.* (2016) 53:313–25. 10.3109/10408363.2016.1150252 26856991

[5] Harris EK Yasaka T. On the calculation of a “reference change” for comparing two consecutive measurements. *Clin Chem.* (1983) 29:25–30. 10.1093/clinchem/29.1.25 6848276

[6] Fraser CG. Inherent biological variation and reference values. *Clin Chem Lab Med.* (2004) 42:758–64. 10.1515/CCLM.2004.128 15327011

[7] Aarsand AK Røraas T Bartlett WA Cos̨kun A Carobene A Fernandez-Calle P Harmonization initiatives in the generation, reporting and application of biological variation data. *Clin Chem Lab Med.* (2018) 56:1629–36. 10.1515/cclm-2018-0058 29596051

[8] Aarsand AK Fernandez-Calle P Webster C Coskun A Gonzales-Lao E Diaz-Garzon J *The EFLM Biological Variation Database.* (2022). Available online at: https://biologicalvariation.eu/ (accessed July 25, 2022).

[9] Aarsand AK Røraas T Fernandez-Calle P Ricos C Díaz-Garzón J Jonker N The biological variation data critical appraisal checklist: a standard for evaluating studies on biological variation. *Clin Chem.* (2018) 64:501–14. 10.1373/clinchem.2017.281808 29222339

[10] Badrick T Turner P. The uncertainty of the eGFR. *Indian J Clin Biochem.* (2013) 28:242–7. 10.1007/s12291-012-0280-1 24426218 PMC3689324

[11] Farrance I Frenkel R. Uncertainty of measurement: a review of the rules for calculating uncertainty components through functional relationships. *Clin Biochem Rev.* (2012) 33:49–75. 22896744 PMC3387884

[12] Rowe C Sitch AJ Barratt J Brettell EA Cockwell P Dalton RN Biological variation of measured and estimated glomerular filtration rate in patients with chronic kidney disease. *Kidney Int.* (2019) 96:429–35. 10.1016/j.kint.2019.02.021 31084924

[13] Carobene A Marino I Cos̨kun A Serteser M Unsal I Guerra E The EuBIVAS project: within- and between-subject biological variation data for serum creatinine using enzymatic and alkaline picrate methods and implications for monitoring. *Clin Chem.* (2017) 63:1527–36. 10.1373/clinchem.2017.275115 28720681

[14] Hilderink JM van der Linden N Kimenai DM Litjens EJR Klinkenberg LJJ Aref BM Biological variation of creatinine, cystatin C, and eGFR over 24 Hours. *Clin Chem.* (2018) 64:851–60. 10.1373/clinchem.2017.282517 29483105

[15] Fraser CG. Reference change values. *Clin Chem Lab Med.* (2011) 50:807–12. 10.1515/cclm.2011.733 21958344

[16] Curto JD Pinto JC. The coefficient of variation asymptotic distribution in the case of non-iid random variables. *J Appl Stat.* (2009) 36:21–32. 10.1080/02664760802382491

[17] Viechtbauer W Cheung MW. Outlier and influence diagnostics for meta-analysis. *Res Synth Methods.* (2010) 1:112–25. 10.1002/jrsm.11 26061377

[18] R Core Team. *R: A Language and Environment for Statistical Computing.* Vienna: R Foundation for Statistical Computing (2020).

[19] Meijers WC van der Velde AR Muller Kobold AC Dijck-Brouwer J Wu AH Jaffe A Variability of biomarkers in patients with chronic heart failure and healthy controls. *Eur J Heart Fail.* (2017) 19:357–65. 10.1002/ejhf.669 27766733 PMC5347881

[20] Reinhard M Erlandsen EJ Randers E. Biological variation of cystatin C and creatinine. *Scand J Clin Lab Invest.* (2009) 69:831–6. 10.3109/00365510903307947 19929276

[21] Carobene A Graziani MS Lo Cascio C Tretti L Cremonese E Yabarek T Age dependence of within-subject biological variation of nine common clinical chemistry analytes. *Clin Chem Lab Med.* (2012) 50:841–4. 10.1515/cclm-2011-0868 22628327

[22] Larsson A Akerstedt T Hansson LO Axelsson J. Circadian variability of cystatin C, creatinine, and glomerular filtration rate (GFR) in healthy men during normal sleep and after an acute shift of sleep. *Chronobiol Int.* (2008) 25:1047–61. 10.1080/07420520802553614 19005904

[23] Hölzel WG. Intra-individual variation of some analytes in serum of patients with insulin-dependent diabetes mellitus. *Clin Chem.* (1987) 33:57–61. 10.1093/clinchem/33.1.57 3802496

[24] Pineda-Tenor D Laserna-Mendieta EJ Timón-Zapata J Rodelgo-Jiménez L Ramos-Corral R Recio-Montealegre A Biological variation and reference change values of common clinical chemistry and haematologic laboratory analytes in the elderly population. *Clin Chem Lab Med.* (2013) 51:851–62. 10.1515/cclm-2012-0701 23518452

[25] IntHout J Ioannidis JP Rovers MM Goeman JJ. Plea for routinely presenting prediction intervals in meta-analysis. *BMJ Open.* (2016) 6:e010247. 10.1136/bmjopen-2015-010247 27406637 PMC4947751

[26] Biosca C Ricós C Lauzurica R Petersen PH. Biological variation at long-term renal post-transplantation. *Clin Chim Acta.* (2006) 368:188–91. 10.1016/j.cca.2005.12.018 16458873

[27] Fraser CG Hearne CR. Components of variance of some plasma constituents in patients with myocardial infarction. *Ann Clin Biochem.* (1982) 19:431–4. 10.1177/000456328201900608 7159012

[28] Ozturk OG Paydas S Balal M Sahin G Karacor ED Ariyurek SY Biological variations of some analytes in renal posttransplant patients: a different way to assess routine parameters. *J Clin Lab Anal.* (2013) 27:438–43. 10.1002/jcla.21625 24218125 PMC6807446

[29] Winkel P Statland BE Bokelund H. Factors contributing to intra-individual variation of serum constituents: 5. Short-term day-to-day and within-hour variation of serum constituents in healthy subjects. *Clin Chem.* (1974) 20:1520–7. 10.1093/clinchem/20.12.1520 4430129

[30] Page MJ McKenzie JE Bossuyt PM Boutron I Hoffmann TC Mulrow CD The PRISMA 2020 statement: an updated guideline for reporting systematic reviews. *BMJ* (2021) 372:n71 10.1136/bmj.n71 33782057 PMC8005924

[31] Kidney Disease. Improving global outcomes (KDIGO) CKD work group: KDIGO 2012 clinical practice guideline for the evaluation and management of chronic kidney disease. *Kidney Int Suppl.* (2012) 3:1.

[32] National Collaborating Centre for Chronic Conditions. *National Institute for Health and Clinical Excellence: Guidance. Chronic Kidney Disease: National Clinical Guideline for Early Identification and Management in Adults in Primary and Secondary Care.* London: Royal College of Physicians (2008). 21413194

[33] Shlipak MG Tummalapalli SL Boulware LE Grams ME Ix JH Jha V The case for early identification and intervention of chronic kidney disease: conclusions from a kidney disease: improving global outcomes (KDIGO) controversies conference. *Kidney Int.* (2021) 99:34–47. 33127436 10.1016/j.kint.2020.10.012

[34] Baysoy A Karakoyun I Arslan FD Basok BI Colak A Duman C. Biological variation data for kidney function related parameter: serum beta trace protein, creatinine and cystatin C from 22 apparently healthy Turkish subjects. *Clin Chem Lab Med.* (2022) 60:584–92. 10.1515/cclm-2021-0543 34506692

[35] Carobene A Banfi G Locatelli M Vidali M. Within-person biological variation estimates from the European biological variation study (EuBIVAS) for serum potassium and creatinine used to obtain personalized reference intervals. *Clin Chim Acta Int J Clin Chem.* (2021) 523:205–7. 10.1016/j.cca.2021.09.018 34571007

[36] Kerschbaum J Rudnicki M Dzien A Dzien-Bischinger C Winner H Heerspink HL Intra-individual variability of eGFR trajectories in early diabetic kidney disease and lack of performance of prognostic biomarkers. *Sci Rep.* (2020) 10:19743. 10.1038/s41598-020-76773-0 33184434 PMC7665005

[37] Levey AS Bosch JP Lewis JB Greene T Rogers N Roth D. A more accurate method to estimate glomerular filtration rate from serum creatinine: a new prediction equation. Modification of diet in renal disease study group. *Ann Intern Med.* (1999) 130:461–70. 10.7326/0003-4819-130-6-199903160-00002 10075613

[38] Levey AS Stevens LA Schmid CH Zhang YL Castro AF III Feldman HI A new equation to estimate glomerular filtration rate. *Ann Intern Med.* (2009) 150:604–12. 10.7326/0003-4819-150-9-200905050-00006 19414839 PMC2763564

[39] Bandaranayake N Ankrah-Tetteh T Wijeratne S Swaminathan R. Intra-individual variation in creatinine and cystatin C. *Clin Chem Lab Med.* (2007) 45:1237–9. 10.1515/CCLM.2007.256 17848122

[40] Biosca C Ricós C Jiménez CV Lauzurica R Galimany R. Model for establishing biological variation in non-healthy situations: renal posttransplantation data. *Clin Chem.* (1997) 43:2206–8. 10.1093/clinchem/43.11.2206 9365419

[41] Carter JL Parker CT Stevens PE Eaglestone G Knight S Farmer CK Biological variation of plasma and urinary markers of acute kidney injury in patients with chronic kidney disease. *Clin Chem.* (2016) 62:876–83. 10.1373/clinchem.2015.250993 27026288

[42] Costongs GM Janson PC Bas BM Hermans J van Wersch JW Brombacher PJ. Short-term and long-term intra-individual variations and critical differences of clinical chemical laboratory parameters. *J Clin Chem Clin Biochem.* (1985) 23:7–16. 10.1515/cclm.1985.23.7.405 3981091

[43] Dimitri G Bolner A Gheller A. Variabilità biologica ed analitica e differenza critica di 21 analiti “comuni”. *Biochim Clin.* (1992) 16:401–3.

[44] Fraser CG Williams P. Short-term biological variation of plasma analytes in renal disease. *Clin Chem.* (1983) 29:508–10. 10.1093/clinchem/29.3.508 6825264

[45] Fraser CG Cummings ST Wilkinson SP Neville RG Knox JD Ho O Biological variability of 26 clinical chemistry analytes in elderly people. *Clin Chem.* (1989) 35:783–6. 10.1093/clinchem/35.5.783 2720971

[46] Gallagher SK Johnson LK Milne DB. Short- and long-term variability of selected indices related to nutritional status. II. Vitamins, lipids, and protein indices. *Clin Chem.* (1992) 38(8 Pt 1):1449–53. 10.1093/clinchem/38.8.1449 1643714

[47] González-Revaldería J García-Bermejo S Menchén-Herreros A Fernandez-Rodriguez E. Towards narrower analytical goals in routine laboratory work. *Clin Chem.* (1991) 37:596. 10.1093/clinchem/37.4.596 2015690

[48] Gowans EM Fraser CG. Biological variation of serum and urine creatinine and creatinine clearance: ramifications for interpretation of results and patient care. *Ann Clin Biochem.* (1988) 25(Pt 3):259–63. 10.1177/000456328802500312 3400982

[49] Keevil BG Kilpatrick ES Nichols SP Maylor PW. Biological variation of cystatin C: implications for the assessment of glomerular filtration rate. *Clin Chem.* (1998) 44:1535–9. 10.1093/clinchem/44.7.1535 9665434

[50] Matsubara A Ichihara K Fukutani S. Determination of reference intervals for 26 commonly measured biochemical analytes with consideration of long-term within-individual variation. *Clin Chem Lab Med.* (2008) 46:691–8. 10.1515/CCLM.2008.140 18598206

[51] Nunes LA Brenzikofer R de Macedo DV. Reference change values of blood analytes from physically active subjects. *Eur J Appl Physiol.* (2010) 110:191–8. 10.1007/s00421-010-1493-8 20446091

[52] Qi Z Chen Y Zhang L Ma X Wang F Cheng Q Biological variations of thirteen plasma biochemical indicators. *Clin Chim Acta.* (2016) 452:87–91. 10.1016/j.cca.2015.11.008 26561925

[53] Ravn B Larsson A Mårtensson J Martling CR Bell M. Intra-day variability of cystatin C, creatinine and estimated GFR in intensive care patients. *Clin Chim Acta.* (2016) 460:1–4. 10.1016/j.cca.2016.06.014 27315745

[54] Rosano TG Brown HH. Analytical and biological variability of serum creatinine and creatinine clearance: implications for clinical interpretation. *Clin Chem.* (1982) 28:2330–1. 10.1093/clinchem/28.11.2330 7127791

[55] Statland BE Winkel P Bokelund H. Factors contributing to intra-individual variation of serum constituents. 1. Within-day variation of serum constituents in healthy subjects. *Clin Chem.* (1973) 19:1374–9. 10.1093/clinchem/19.12.1374 4757366

[56] Toffaletti JG McDonnell EH. Variation of serum creatinine, cystatin C, and creatinine clearance tests in persons with normal renal function. *Clin Chim Acta.* (2008) 395:115–9. 10.1016/j.cca.2008.05.020 18573244

[57] Waikar SS Rebholz CM Zheng Z Hurwitz S Hsu CY Feldman HI Biological variability of estimated GFR and albuminuria in CKD. *Am J Kidney Dis.* (2018) 72:538–46. 10.1053/j.ajkd.2018.04.023 30031564 PMC6469385

[58] Wang S Zhao M Su Z Mu R. Annual biological variation and personalized reference intervals of clinical chemistry and hematology analytes. *Clin Chem Lab Med.* (2022) 60:606–17. 10.1515/cclm-2021-0479 34773728

[59] Williams GZ Widdowson GM Penton J. Individual character of variation in time-series studies of healthy people: II. Differences in values for clinical chemical analytes in serum among demographic groups, by age and sex. *Clin Chem.* (1978) 24:313–20. 10.1093/clinchem/24.2.313 627063

[60] Young DS Harris EK Cotlove E. Biological and analytic components of variation in long-term studies of serum constituents in normal subjects. IV. Results of a study designed to eliminate long-term analytic deviations. *Clin Chem.* (1971) 17:403–10. 10.1093/clinchem/17.5.403 5573406

