# Supplementary figures and images for: Biological variation and reference change value of the estimated glomerular filtration rate in humans: A systematic review and meta-analysis
Source: Front Med (Lausanne). 2022 Oct 6;9:1009358. doi: 10.3389/fmed.2022.1009358 (PMC9583397; doi:10.3389/fmed.2022.1009358)

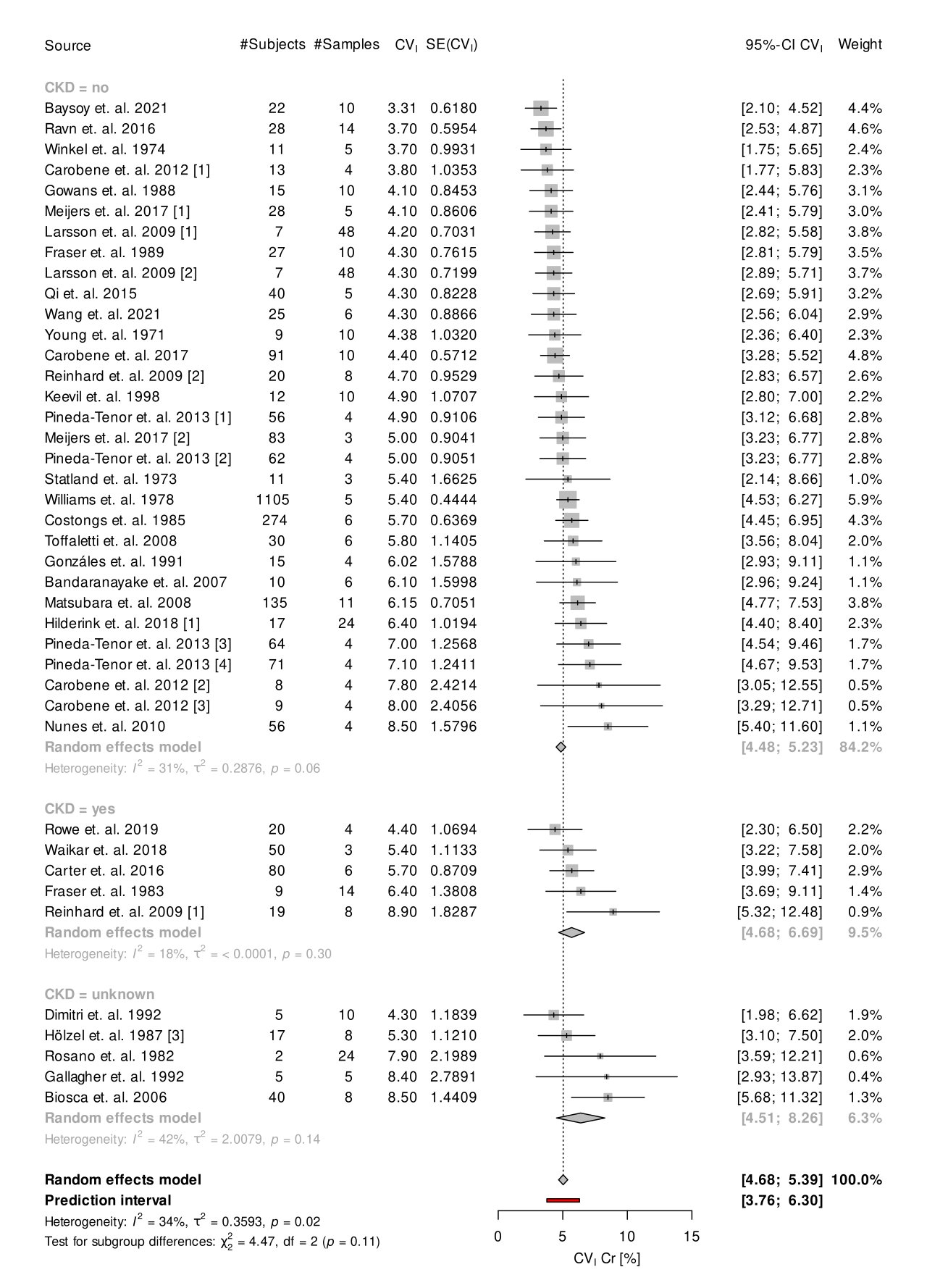

Supplement: Supplementary Image 1 — Subgroup analysis for presence of CKD. [file Image_1.JPEG]
