# Supplementary material for: Biological variation and reference change value of the estimated glomerular filtration rate in humans: A systematic review and meta-analysis
Source: Front Med (Lausanne). 2022 Oct 6;9:1009358. doi: 10.3389/fmed.2022.1009358 (PMC9583397; doi:10.3389/fmed.2022.1009358)
Supplement: Supplementary file 2 [file Table_1.DOCX]

Search term:

("creatinine/blood"[MeSH Terms] OR "Serum Creatinine"[ti] OR "Creatinine"[ti] OR "eGFR"[ti] OR "glomerular filtration rate"[ti] OR "estimated glomerular filtration rate"[ti] OR "GFR" OR "analytes"[ti] OR "clinical chemistry analytes"[ti]) AND ("biological variability"[ti] OR "Biological Variation"[ti] OR "biological variations"[ti] OR "reference change value"[ti] OR "within subject biological variation"[ti] OR "coefficient of variation"[ti] OR "intraindividual variation"[ti] OR "variation"[ti] OR "variability"[ti] OR "coefficient of variability"[ti] OR "Biological Variation, Individual"[Majr])
