# Supplementary material for: Biological variation and reference change value of the estimated glomerular filtration rate in humans: A systematic review and meta-analysis
Source: Front Med (Lausanne). 2022 Oct 6;9:1009358. doi: 10.3389/fmed.2022.1009358 (PMC9583397; doi:10.3389/fmed.2022.1009358)
Supplement: Supplementary Table 2 — Main study characteristics. [file Table_2.DOCX]

|  |  | BIVAC Score Quality Items | | | | | | | | | | | | | | |  |  |  |  |  |  |  |
| --- | --- | --- | --- | --- | --- | --- | --- | --- | --- | --- | --- | --- | --- | --- | --- | --- | --- | --- | --- | --- | --- | --- | --- |
| study | year | Q1 | Q 2 | Q 3 | Q 4 | Q 5 | Q 6 | Q 7 | Q 8 | Q 9 | Q  10 | Q  11 | Q  12 | Q  13 | Q  14 | total score | method | no. of subjects | males | females | health status | no. of samples per patient | cvi |
| Bandaranayake et al. | 2007 | A | A | A | A | A | B | B | C | B | C | B | A | C | A | C | jaffe | 10 | 4 | 6 | healthy | 6 | 6.1 |
| Baysoy et al. | 2021 | A | A | A | A | A | A | A | A | A | A | A | A | A | A | A | jaffe | 22 | 13 | 9 | healthy | 10 | 3.3 |
| Biosca et al. | 1997 | A | A | A | A | A | B | A | A | A | C | A | A | A | B | C | unknown | 19 | 12 | 7 | non healthy | 33 | 11.8 |
| Biosca et al. | 2006 | A | A | A | A | A | B | A | A | B | C | A | C | A | A | C | jaffe | 40 | 26 | 14 | non healthy | 8 | 8.5 |
| Carobene et al. | 2012 | A | A | A | A | A | A | B | B | B | C | A | C | A | A | C | jaffe | 8 | 4 | 4 | healthy | 4 | 7.8 |
| Carobene et al. | 2012 | A | A | A | A | A | A | B | B | B | C | A | C | A | A | C | jaffe | 9 | 4 | 5 | healthy | 4 | 8.0 |
| Carobene et al. | 2012 | A | A | A | A | A | A | B | B | B | C | A | C | A | A | C | jaffe | 13 | 6 | 7 | healthy | 4 | 3.8 |
| Carobene et al. | 2017 | A | A | A | A | A | A | A | A | A | A | A | A | A | A | A | enzymatic | 91 | 38 | 53 | healthy | 10 | 4.4 |
| Carter et al. | 2016 | A | A | A | A | A | A | B | A | A | A | A | A | B | A | B | enzymatic | 80 | 47 | 33 | non healthy | 6 | 5.7 |
| Costongs et al. | 1985 | A | A | A | C | A | B | A | C | B | C | B | A | C | A | C | unknown | 274 | 148 | 126 | healthy | 6 | 5.7 |
| Dimitri et al. | 1992 | A | A | A | A | A | A | B | C | B | C | A | A | C | B | C | jaffe | 5 |  |  | healthy | 10 | 4.3 |
| Fraser et al. [1] | 1983 | A | A | A | A | A | A | B | B | B | C | A | A | A | A | C | jaffe | 9 |  |  | non healthy | 14 | 6.4 |
| Fraser et al. [2] | 1989 | A | A | A | C | A | A | A | B | B | C | A | A | A | A | C | unknown | 27 | 14 | 13 | healthy | 10 | 4.3 |
| Fraser et al. [3] | 1982 | A | A | A | A | A | A | A | B | B | C | A | A | A | A | C | jaffe | 20 |  |  | healthy | 31 | 13.4 |
| Gallagher et al. | 1992 | A | A | A | A | A | B | B | C | B | A | A | A | B | A | C | jaffe | 5 | 0 | 5 | healthy | 5 | 8.4 |
| Gonzáles et al. | 1991 | A | A | A | A | A | A | A | B | B | C | B | A | A | A | C | jaffe | 15 | 5 | 10 | healthy | 4 | 6.0 |
| Gowans et al. | 1988 | A | A | A | A | A | A | B | B | B | C | B | A | C | A | C | jaffe | 15 | 7 | 8 | healthy | 10 | 4.1 |
| Hilderink et al. [1] | 2018 | A | A | A | A | A | A | A | A | A | A | A | A | A | A | A | enzymatic | 17 | 14 | 3 | non healthy | 24 | 6.4 |
| Hilderink et al. [2] | 2018 | A | A | A | A | A | A | A | A | A | A | A | A | A | A | A | enzymatic | 19 | 13 | 6 | non healthy | 24 | 2.5 |
| Hölzel et al. [1] | 1987 | A | A | A | A | A | A | A | C | A | A | B | A | C | A | C | jaffe | 10 | 10 | 0 | healthy | 8 | 2.6 |
| Hölzel et al. [2] | 1987 | A | A | A | A | A | A | A | C | A | A | B | A | C | A | C | jaffe | 14 | 0 | 14 | healthy | 8 | 2.8 |
| Hölzel et al. [3] | 1987 | A | A | A | A | A | A | A | C | A | A | B | A | C | A | C | jaffe | 17 | 8 | 9 | non healthy | 8 | 5.3 |
| Keevil et al. | 1998 | A | A | A | A | A | A | B | C | B | C | B | A | C | A | C | jaffe | 12 | 7 | 5 | healthy | 10 | 4.9 |
| Larsson et al. [1] | 2009 | A | A | A | A | A | B | B | C | B | C | A | C | C | B | C | jaffe | 7 | 7 | 0 | healthy | 48 | 4.2 |
| Larsson et al. [2] | 2009 | A | A | A | A | A | B | B | C | B | C | A | C | C | B | C | jaffe | 7 | 7 | 0 | healthy | 48 | 4.3 |
| Matsubara et al. | 2008 | A | A | A | C | B | A | B | C | B | C | A | C | A | A | C | unknown | 135 | 60 | 75 | healthy | 11 | 6.2 |
| Meijers et al. [1] | 2017 | A | A | C | C | B | A | A | C | A | C | A | A | A | A | C | jaffe | 28 | 14 | 14 | healthy | 5 | 4.1 |
| Meijers et al. [2] | 2017 | A | A | C | C | B | A | A | C | A | C | A | A | A | A | C | jaffe | 83 | 79 | 4 | non healthy | 3 | 5.0 |
| Nunes et al. | 2010 | A | A | A | A | A | B | B | A | A | A | A | A | A | A | B | unknown | 56 | 56 | 0 | healthy | 4 | 8.5 |
| Ozturk et al. | 2013 | B | A | A | A | A | B | A | A | B | A | A | A | B | B | B | jaffe | 70 | 49 | 21 | non healthy | 6 | 9.2 |
| Pineda-Tenor et al. [1] | 2013 | A | A | A | A | A | B | B | C | B | A | A | A | B | A | C | jaffe | 56 | 56 | 0 | healthy | 4 | 4.9 |
| Pineda-Tenor et al. [2] | 2013 | A | A | A | A | A | B | B | C | B | A | A | A | B | A | C | jaffe | 62 | 0 | 62 | healthy | 4 | 5.0 |
| Pineda-Tenor et al. [3] | 2013 | A | A | A | A | A | B | B | C | B | A | A | A | B | A | C | jaffe | 64 | 0 | 64 | healthy | 4 | 7.0 |
| Pineda-Tenor et al. [4] | 2013 | A | A | A | A | A | B | B | C | B | A | A | A | B | A | C | jaffe | 71 | 71 | 0 | healthy | 4 | 7.1 |
| Qi et al. | 2015 | A | A | A | A | A | A | B | C | B | C | A | A | C | A | C | unknown | 40 | 21 | 19 | healthy | 5 | 4.3 |
| Ravn et al. | 2016 | A | A | A | A | B | C | A | C | B | C | C | C | C | B | C | unknown | 28 | 13 | 15 | non healthy | 14 | 3.7 |
| Reinhard et al. [1] | 2009 | A | A | A | A | A | A | C | B | B | C | A | A | B | A | C | enzymatic | 19 | 11 | 8 | non healthy | 8 | 8.9 |
| Reinhard et al. [2] | 2009 | A | A | A | A | A | A | B | B | B | C | A | A | B | A | C | enzymatic | 20 | 7 | 13 | healthy | 8 | 4.7 |
| Rosano et al. | 1982 | A | A | A | A | A | A | B | C | B | C | C | A | A | A | C | jaffe | 2 | 2 | 0 | healthy | 24 | 7.9 |
| Rowe et al. | 2019 | A | A | A | A | A | A | A | A | A | A | A | A | A | A | A | enzymatic | 20 | 10 | 10 | non healthy | 4 | 4.4 |
| Statland et al. | 1973 | A | A | A | A | A | A | B | C | B | A | B | A | C | A | C | jaffe | 11 | 11 | 0 | healthy | 3 | 5.4 |
| Toffaletti et al. | 2008 | A | A | B | A | B | C | B | C | B | C | C | C | C | A | C | enzymatic | 30 | 15 | 15 | healthy | 6 | 5.8 |
| Waikar et al. | 2018 | A | A | A | A | A | B | A | C | A | C | C | A | C | A | C | enzymatic | 50 | 28 | 22 | non healthy | 3 | 5.4 |
| Wang et al. | 2021 | A | A | A | A | A | B | A | B | B | C | B | A | B | A | C | enzymatic | 25 | 8 | 17 | healthy | 6 | 4.3 |
| Williams et al. | 1978 | A | A | A | C | A | C | B | C | B | C | C | C | C | A | C | unknown | 1105 | 628 | 477 | healthy | 5 | 5.4 |
| Winkel et al. [1] | 1974 | A | A | A | A | A | A | B | C | B | C | A | C | C | A | C | unknown | 11 | 11 | 0 | healthy | 5 | 3.7 |
| Winkel et al. [2] | 1976 | A | A | A | A | A | A | B | C | B | C | A | C | C | A | C | unknown | 10 | 0 | 10 | healthy | 6 | 1.2 |
| Young et al. | 1971 | A | A | A | A | A | A | B | C | B | C | A | A | C | A | C | jaffe | 9 | 9 | 0 | healthy | 10 | 4.4 |
